# Low-field electron mobility of InSb nanowires: Numerical efforts to larger cross sections

**DOI:** 10.1038/s41598-017-02536-z

**Published:** 2017-05-31

**Authors:** Wei Feng, Chen Peng, Shuang Li, Xin-Qi Li

**Affiliations:** 1 Department of Physics, Tianjin University, Tianjin, 300072 China; 2 Department of Physics, Renmin University of China, Beijing, 100872 China; 3 Center for Advanced Quantum Studies and Department of Physics, Beijing Normal University, Beijing, 100875 China

## Abstract

Within the framework of Boltzmann equation, we present a **k** · **p** theory based study for the low-field mobilities of InSb nanowires (InSb NWs) with relatively large cross sectional sizes (with diameters up to 51.8 nm). For such type of large size nanowires, the intersubband electron-phonon scattering is of crucial importance to affect the scattering rate and then the mobility. In our simulation, the lowest 15 electron subbands and 50 transverse modes of phonons are carefully accounted for. We find that, up to the 51.84 nm diameter, the mobility monotonously increases with the diameter, not yet showing any saturated behavior. We also find that, while the bulk InSb mobility is considerably higher than the bulk Si, the small size (e.g. ~3 nm diameter) nanowires from both materials have similar magnitude of mobilities. This implies, importantly, that the mobility of the InSb NWs would decrease faster than the SiNWs as we reduce the cross sectional size of the nanowires.

## Introduction

Mobility is one of the most important figures to affect material properties and device applications. For nano-electronic purpose, device miniaturization usually requires engineering the bulk materials to smaller sizes or to lower dimensions. In this context, how the mobility changes with the size and dimensionality is a crucial problem.

For theoretical investigations, the first intrinsic mechanism of mobility is the electron phonon scattering. This requires to know the full knowledge of electron and phonon states, and to properly account for the summation over a large number of discrete phonon modes and electron subbands under the restriction of energy conservation. We notice that so far only very limited theoretical calculations have been reported. Among them, representative examples are the mobility calculations of Si nanowires (SiNWs), up to diameters of a few nanometers^1–6^.

In the past years the InSb material and InSb NWs^7, 8^ have attracted intensive attention and promise sound applications in high-speed field effect and new-concept quantum devices, owing to the high mobility (up to 77000 cm^2^ V^−1^ s^−1^ at room temperature) and large Landé *g* factor. A distinguished example may be the demonstration of Majorana fermion in the hybrid system by contacting the InSb NW with a superconductor^9–12^.

In this work we present a state-of-the-art calculation for the phonon limited electron mobility of the InSb NWs. In particular, we make efforts to extend theoretical studies to nanowires with relatively large diameters, for instance, up to 51.8 nm. For this purpose, instead of the first-principle or the tight-banding schemes, we employ the eight-band **k** · **p** theory to calculate the electronic structures of the InSb NWs, with tolerable computational expenses^13–21^. The **k** · **p** theory should be appropriate for the *low*-*field mobility* calculation, for which the acoustic phonon scattering is also the dominant *intrinsic mechanism*. For the phonon modes, we employ the continuum media model. We apply the deformation-potential theory to address the electron-phonon interaction and apply the Boltzmann equation to formulate the low-field mobility calculation.

## Results

### Electron Energy Subbands

We employ the eight-band **k** · **p** theory for the calculation of electronic states, which can provide reliable result around a given high-symmetry point **k**_**0**_ (usually the Γ point). In this method, one conventionally denotes the eight *known* states at **k**_**0**_ by $|J,{J}_{z}\rangle \equiv |n\rangle$, where *J* and *J*_*z*_ are the total angular momentum and its components which characterize, respectively, the conduction-band electron (*J* = 1/2 and *J*_*z*_ = ±1/2), the valence-band heavy (*J*_*z*_ = ±3/2) and light (*J*_*z*_ = ±1/2) holes with *J* = 3/2, and the spin-orbit coupling split states in valence band with *J*_*z*_ = ±1/2.

To apply the **k** · **p** theory to confined system as the nanowire with circular cross section, it would be convenient to use the eigen-functions solved from a cylindrical wire to constitute the representation basis of the envelope function, which plays a role to modulate the periodic kernel function of the bulk Bloch state. This basis function, ${\psi }_{L,m}({k}_{z},{\bf{r}})=\langle {\bf{r}}|{k}_{z},L,m\rangle$, is simply given by

1 $${\psi }_{L,m}({k}_{z},{\bf{r}})=\frac{1}{\sqrt{l}}{e}^{i{k}_{z}z}\frac{1}{\sqrt{\pi }}\frac{1}{R{J}_{L+1}({\alpha }_{m}^{L})}{J}_{L}(\frac{{\alpha }_{m}^{L}}{R}r){e}^{iL\phi },$$

where *l* and *R* are respectively the length and cross section radius of the nanowire. *k*_*z*_ is the wave vector along the *z*-axis. The lateral quantization of the wave function is characterized the quantum numbers *L* and *m*. The *J*_*L*_(*x*) is the *L*-th order Bessel function of the first kind, with ${\alpha }_{m}^{L}$ of its *m*-th root. Here we have denoted the polar coordinates in the circular cross section by (*r*, *φ*).

Using the basis set $\{|n\rangle \otimes |{k}_{z},L,m\rangle \}$, the electron eigen-energies and states can be solved from

2 $$\sum _{n^{\prime} ,L^{\prime} ,m^{\prime} }\,\langle {k}_{z},L,m|{ {\mathcal H} }_{n,n^{\prime} }|{k}_{z},L^{\prime} ,m^{\prime} \rangle {C}_{n^{\prime} ,L^{\prime} ,m^{\prime} }^{(\nu )}={E}_{\nu }({k}_{z}){C}_{n,L,m}^{(\nu )},$$

where ${ {\mathcal H} }_{n,n^{\prime} }$ is the **k** · **p** Hamiltonian matrix element of bulk material. Owing to lateral confinement, the components of the wave vector *k*_*x*_ and *k*_*y*_ in ${ {\mathcal H} }_{n,n^{\prime} }$ become now the momentum operators, acting on the spatial coordinates of the lateral wavefunction (*ψ*_*L*,*m*_) in the cross section of the nanowire. Corresponding to the eigen-energy *E*_*ν*_(*k*_*z*_), the eigen-state reads $|{{\rm{\Psi }}}_{\nu }\rangle ={\sum }_{n,L,m}\,{C}_{n,L,m}^{(\nu )}|n\rangle \otimes |{k}_{z},L,m\rangle$, which will be used in this work to calculate the electron-phonon scattering rate.

In the **k** · **p** theory, there exits the so-called *spurious solution* problem, caused by the *incompleteness* of the basis functions included in practical computation. A couple of schemes were proposed to partially overcome this difficulty, such as discarding terms in the Hamiltonian^14^ or rejecting the unphysical large *k* solutions^15, 16^. In this work we adopt the method proposed in ref. 17, by modifying parameters to make the *k* ^2^ terms be zero in the conduction-band matrix elements in ${ {\mathcal H} }_{n,n^{\prime} }$, meanwhile properly fitting the conduction-band effective mass. After this type of treatment/modification to the Hamiltonian matrix, no spurious solution will appear and one is able to recover all the other effective masses obtained by experiment.

**Figure 1:**
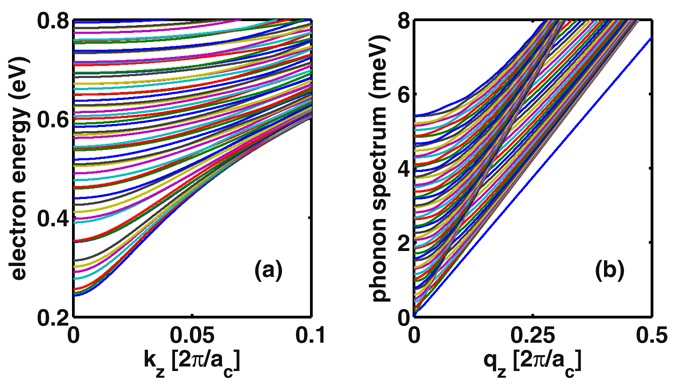
(**a**) Conduction subbands of InSb nanowire along the [001] direction with diameter 51.8 nm, based on the **k** · **p** theory calculation. (**b**) Phonon spectrum of the same InSb nanowire calculated from a continuum media model. In both plots, *a*_*c*_ is the lattice constant.

Applying the eight-band **k** · **p** theory to the zinc-blende InSb^18^ and using the material parameters from ref. 22, we are able to calculate the electronic states of the InSb nanowires up to relatively large sizes. In Fig. 1(a) we show the result of a couple of conduction subbands in the [001] direction, for an InSb NW with diameter 51.8 nm. We see that with the increase of the size of the nanowire, the energy spacing between the subbands decreases. This will make the intersubband scattering be of crucial importance in the mobility calculation for large size nanowires. In this work we will make particular efforts to account for this issue.

### Phonon Spectrum

For the acoustic phonons, we apply the continuous medium model to solve the vibrational modes under a freestanding boundary condition (FSBC). After some algebras, the problem is reduced to the following Pochhammer-Chree equation^23^

3 $$\begin{array}{c}\frac{2{q}_{l}}{R}({q}_{t}^{2}+{q}_{z}^{2}){J}_{1}({q}_{l}R){J}_{1}({q}_{t}R)-{({q}_{t}^{2}-{q}_{z}^{2})}^{2}{J}_{0}({q}_{l}R){J}_{1}({q}_{t}R)\\ \quad -4{q}_{z}^{2}{q}_{l}{q}_{t}{J}_{1}({q}_{l}R){J}_{0}({q}_{t}R)=0.\end{array}$$

*J*_0_ and *J*_1_ are the Bessel functions. *q*_*l*_ and *q*_*t*_ are defined through ${q}_{l,t}^{2}=\tfrac{{\omega }^{2}}{{v}_{l,t}^{2}}-{q}_{z}^{2}$, where *ω* and *q*_*z*_ are respectively the vibration frequency and the longitudinal wave vector (along the axial direction of the nanowire), while *v*_*l*_ and *v*_*t*_ are the speeds of the longitudinal and transverse waves of bulk material. For InSb in the [100] direction, we take *v*_*l*_ = 3.4 × 10^5^ cm/s and *v*_*t*_ = 2.3 × 10^5^ cm/s, following ref. 24.

In Fig. 1(b), we display the dispersion relation of the lowest 50 transverse phonon modes, for an InSb NW with diameter of 51.8 nm. This number of transverse modes will be included into the electron phonon scattering in our mobility calculation. In particular, we remark here that the *circular* cross sectional nanowires under FSBC can support the existence of transverse vibrational mode with zero frequency. This property will have dramatic effect on the momentum relaxation rate of electrons.

### Momentum Relaxation Rate

**Figure 2:**
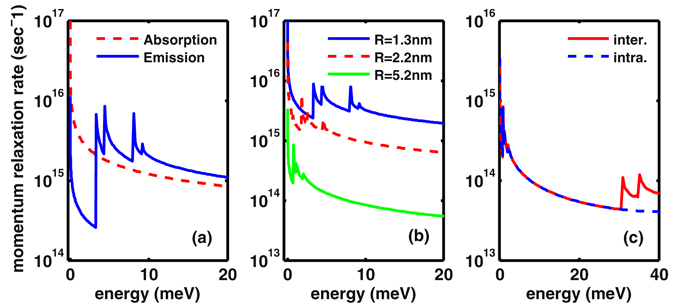
(**a**) Momentum relaxation rate (as a function of the initial energy of the electron) calculated for an InSb nanowire with radius 1.3 nm. The rates owing to phonon emission and absorption are presented separately and only phonon emission peaks appear in the rate. (**b**) The summed total relaxation rates for phonon emission and absorption, for a couple of cross sectional sizes. (**c**) Effect of inclusion of the intersubband scattering for an InSb nanowire with radius 5.2 nm (solid red curve). Compared to the intrasubband-only-scattering (blue dashed curve), magnitude change and additional peaks appear in the relaxation rate of electron with higher energies (e.g. over 30 meV in this plot). All the results in (**a**–**c**) are calculated under temperature 300 K.

For the dominant intrinsic mechanism at low fields, we employ the deformation potential (DP) model for the electron-acoustic-phonon scattering^25, 26^. The Hamiltonian reads ${H}_{ep}={E}_{a}\nabla \cdot {\bf{u}}$, where **u** is the lattice displacement (deformation) and *E*_*a*_ is the DP constant. For InSb, *E*_*a*_ = 5.08 eV, from ref. 22. Applying the Fermi’s golden rule, one can derive an expression for the momentum relaxation rate (see the ‘Method’ part for some details). In numerical implementation, particular attention should be paid to the large number of mode summations under the restriction of energy conservation. We show in Fig. 2 several representative results.

For thin nanowires with small cross section, it is a good approximation to consider electrons populated mainly on the lowest subband and the electron phonon scattering dominantly within the same subband (i.e., intrasubband scattering). In Fig. 2(a) we exemplify the result of an InSb nanowire with radius 1.3 nm. In this plot we separately present the rates from phonon emission and absorption, and find that only phonon emission peaks appear in the rate with the increase of the initial energy of the electron. Each peak indicates that a more transverse mode is newly involved into the scattering. Notably, the ‘half peak’ near zero frequency is resulted from scattering with the transverse zero-mode mentioned above in Fig. 1(b).

In Fig. 2(b) we plot the summed total relaxation rate caused by phonon emission and absorption, for a couple of cross sectional sizes. As already revealed from Fig. 2(a), we know that the peaks in the total rate are resulted from phonon emissions. With the increase of the nanowire sizes, the peaks move to lower frequencies, while at the same time the scattering rate is reduced, owing to the lower energies of the phonon modes, being thus *less efficient* to take away the energy of the electron.

From simple consideration, we expect that the intersubband scattering will become important with the size increase of the nanowires. Indeed, as shown in Fig. 2(c) for an InSb NW with radius 5.2 nm, the effect of inclusion of the intersubband scattering (solid red curve) is prominent for higher electron energy (i.e. over 30 meV), as manifested here in both the magnitude change and additional new peaks. This result clearly tells us that, for larger size nanowires, one must taken into account the inter-subband scattering in the mobility calculation.

### Mobility

With the knowledge of momentum relaxation rate, the electron mobility can be calculated within the framework of Boltzmann equation^27, 28^. First, for the electron in the specific subband *i*, the mobility can be calculated via

4 $${\mu }_{i}=\frac{2e}{{k}_{B}T{m}_{i}^{\ast }}\frac{{\int }_{firstBZ}\,[{E}_{i}({k}_{z})-{E}_{0}]{W}_{i}^{-1}({k}_{z}){f}_{0}({k}_{z})d{k}_{z}}{{\int }_{firstBZ}\,{f}_{0}({k}_{z})d{k}_{z}},$$

where ${m}_{i}^{\ast }$ and *E*_*i*_ are, respectively, the effective mass and energy of electron of subband *i*. *E*_0_ is the lowest energy of conduction band. The integration is over the first Brillouin zone, and the zero-field equilibrium distribution function reads ${f}_{0}({k}_{z})={e}^{-{E}_{i}({k}_{z})/{k}_{B}T}$. ${W}_{i}({k}_{z})={\sum }_{f}\,{W}_{if}({k}_{z})$ is the momentum relaxation rate analyzed above. Next, for the measured mobility in practice, we average all the individuals simply as

5 $$\mu =\frac{1}{\sum _{i}\,{n}_{i}}\sum _{i}\,{\mu }_{i}{n}_{i},$$

where *n*_*i*_ is the population weight of the *i*_th_ subband.

In order to compare the results of finite size nanowire with the bulk mobility, we quote here the mobility formula for the three dimensional (3D) bulk systems derived from the Boltzmann equation and under the deformation potential approximation for the acoustic phonon scattering^27^

6 $$\mu =\frac{2\sqrt{2\pi }\,e{\hslash }^{4}{C}^{3D}}{3{E}_{a}^{2}{({m}_{e}^{\ast })}^{\mathrm{5/2}}{({k}_{B}T)}^{3/2}},$$

where *C* ^3*D*^ is the elastic constant of the 3D material and *E*_*a*_ is the deformation potential. We used the InSb parameters from ref. 22: *E*_*a*_ = 23.3 eV, *C* ^3*D*^ = 684.7 GPa, and ${m}_{e}^{\ast }=0.0135{m}_{0}$.

In our present state-of-the-art calculation, we aim at the electron mobility of the InSb nanowires with relatively large diameter up to 51.8 nm, under temperatures 77 K and 300 K, respectively. In this case, particular attention should be paid to the multiple inter-subband scattering. In our simulation, based on a self-consistent check, we included the lowest 15 electron subbands and 50 transverse modes of phonons. As a comparison, we notice that in refs 1, 2, for the small 3 nm diameter SiNW, the top 3 valence subbands are taken into account for inter-subband scattering in the hole mobility calculation, owing to the relatively small energy spacings.

**Figure 3:**
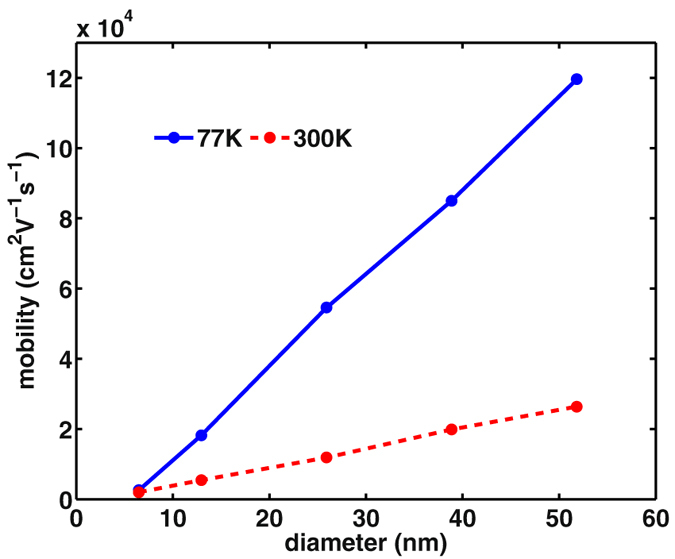
Size dependence of mobility of the InSb nanowires calculated at 77 K (blue dots) and 300 K (red dots).

The key results are displayed in Fig. 3. The easier observation is the temperature effect. We find that only the mobility of small size nanowires has weak dependence of temperature. With the increase of the cross sectional size, the temperature effect becomes more prominent, owing to more phonons excited and involved in the scattering which reduce then the mobility.

More complicated is the size dependence of the mobility. First, the *monotonous increase* of mobility with the diameter, which goes far beyond the size scope explored in refs 1, 2, reveals different features from the small size SiNW. In refs 1, 2 it was found that, at temperature 300 K, the hole mobility of the SiNW with only up to 3.5 nm diameter is higher than the acoustic phonon limited hole mobility of *bulk* Si. This is indeed an unexpected result. Nevertheless, at 77 K, it was found in refs 1, 2 that, comparing the 3 nm diameter SiNW with bulk Si, the hole and electron mobilities are, respectively, 8486 *vs* 11481 cm^2^/Vs for hole, and ~2500 *vs* 23000 cm^2^/Vs for electron.

In our case, we find that up to 51.84 nm diameter the mobility does not show a *saturation* behavior. At temperature 77 K, the computed value of mobility at this size is 1.2 × 10^5^ cm^2^/Vs, which is lower than both the theoretically estimated (acoustic phonon limited) and experimentally measured values of bulk InSb mobility, i.e., 2.8 × 10^7^ and 1.2 × 10^6^ cm^2^/Vs, respectively. For temperature 300 K, the calculated mobility of the 51.84 nm diameter InSb NW is also lower than the bulk mobility, i.e., the theoretical (acoustic phonon limited) result 3.6 × 10^6^ cm^2^/Vs and the measured value 7.7 × 10^4^ cm^2^/Vs. For both temperatures, the reason that the theoretical results are about one order of magnitude higher than the measured values is owing to some more scattering mechanisms involved in real case.

Second, another interesting point to be noted is that, while the bulk InSb mobility – both the theoretical (acoustic phonon limited) and measured values, as mentioned above – is considerably higher than the bulk Si mobility (e.g., at 77 K, 2.3 × 10^4^ cm^2^/Vs), but the InSb nanowire with small diameter has similar mobility as that computed in refs 1, 2 for the 3 nm SiNW, i.e., 2500 cm^2^/Vs. This implies that the mobility of InSb nanowire would decrease faster than the SiNW as we reduce the size of the nanowire.

This feature can be understood as follows. Since the sound speed of Si material (either the longitudinal *v*_*l*_ or the transverse *v*_*t*_) is about 2.5 times faster than the one of the InSb, we know then that the phonon frequency of large size SiNW is roughly 2.5 times higher than the same size InSb NW (and of course for the same wave vector). We also know that higher frequency phonon can dissipate carrier’s energy more efficiently. This may be one of the reasons that the bulk mobility of Si is lower than that of the InSb. However, with the decrease of the size of the nanowire, the lateral confinement will generate high frequency phonons with yet small longitudinal wave-vector. We then expect that the mobility of the InSb NW will decease faster than that of the SiNW with the decrease of the cross sectional size. In particular, for such a small size of ~3 nm diameter, both have similar magnitude.

Finally we remark that the effect of the change of the effective mass has been taken into account in our computation through Eq. (4). With the decrease of the size, the effective mass will increase gradually. For instance, by means of a simple parabolic fit to the energy bands near the high-symmetry point (i.e., for small *k*_*z*_ as illustrated in Fig. 1(a)), we extract the effective masses of the lowest subbands as, respectively, (1.025, 1.034, 1.069, 1.194, 1.5) ${m}_{e}^{\ast }$ for the diameters of (51.8, 38.9, 25.9, 12.9, 6.5) nm, where ${m}_{e}^{\ast }$ is the bulk effective mass of InSb. And, for a given size nanowire, the effective masses of higher subbands will increase with the subband indices, c.f. Fig. 1(a). From Eq. (4) we understand that the mobility is affected by both the scattering rate and the effective mass. Roughly speaking, for large size nanowires, the effective mass is not sensitive to the size, so the mobility is largely affected by the scattering rate. But for small size nanowires, the change of effective mass will more severely affect the mobility. Compared to the results not accounting for the variations of the effective mass, the mobilities shown in Fig. 3 have smaller values (owing to the enhanced effective mass), e.g., for the 6.5 nm nanowire, taking values 2606 *versus* 3890 cm^2^/Vs at 77 K and 2009 *versus* 3148 cm^2^/Vs at 300 K. Nevertheless, this type of changes does not affect the qualitative behavior of the size dependence in the range we investigated.

## Discussion

In this work we present a state-of-the-art calculation for the phonon limited electron mobility of the InSb NWs with diameters up to 51.8 nm. In our calculation we carefully accounted for the complicated intersubband electron-phonon scattering by including the lowest 15 electron subbands and 50 transverse modes of phonons. These efforts extend the existing theoretical studies, e.g., for SiNWs with small diameters about 3 nm^1–6^. One of the reasons to make this be possible is that, rather than the first-principle or the tight-banding methods, we employ the eight-band **k** · **p** theory for the electronic structure calculation with tolerable computational expenses. The **k** · **p** theory should be appropriate for the concerned low-field mobility calculation, for which the acoustic phonon scattering is also the dominant intrinsic mechanism.

Applying the **k** · **p** approach to confined systems^19–21^, one should first obtain the correct **k** · **p** Hamiltonian matrix for the bulk system, c.f. Eq. (2), which contains the *bulk parameters* calculated (or extracted from measurement) at the high-symmetry point. So the crystal variations in the structure of the InSb NWs (e.g., the zinc-blende *versus* wurzite structure) can be accounted for in this theoretical framework. Then, the second step is to convert the wave vectors in the cross section into momentum operators. The new band structure is given by the eigenvalue eq. (2), which provides the essential information of electronic states of the confined system. For the calculation of phonon modes, following the strategy in literature^1, 2, 29^, we simply adopted the DP parameter and sound speed as the bulk values. This approximation is reasonable by noting that these parameters rapidly tend to the bulk values with the increase of diameters, e.g., larger than 5~10 nm^26, 29^. Actually, this approximation has been employed also for the very small size SiNWs^1, 2^ and was supported by the result of the first-principle calculation^26^.

To summarize, our main result shows that, up to relatively large diameters (e.g. 51.84 nm), the mobility of the InSb NWs would monotonously increases with the diameter. This implies a remarkably smaller mobility of InSb NWs than the bulk material. We also find that, while the bulk InSb mobility is considerably higher than the bulk Si, the small size (e.g. ~3 nm diameter) InSb and Si nanowires have similar mobilities. This implies, importantly, that the mobility of the InSb NWs would decrease faster than the SiNWs as we reduce the cross sectional size of the nanowires.

The result in Fig. 3 is only the acoustic phonon limited mobility, which surely overestimates the values of the mobility. In real nanowires, beside the intrinsic phonon scattering, there are also other scattering mechanisms to affect the mobility, among them including such as the neutral and charged impurities inside the nanowire and very importantly, the surface roughness and non-idealities. Further studies should consider these scattering mechanisms, despite that it seems difficult to reliably model them owing to their strong dependence on many real growth conditions. With respect to the temperature dependence, the thermal-excitation-related phonon scattering should be more relevant. Other mechanisms are insensitive to temperatures, as long as the temperature does not affect the impurity and roughness configurations. Within the intrinsic mechanism of phonon scattering, our present study provides an insight into the mobility variation behavior of the specific InSb NWs with cross sectional sizes, beyond most existing calculations, despite further space remaining for future explorations. It would be very interesting (but quite challenging) to extend the study to larger sizes to probe the transition to bulk behavior. With the increase of the diameters, the huge enhancement of both the electron and phonon subbands would make the numerical calculations intractable.

## Methods

Under the deformation potential model for the electron-acoustic-phonon interaction, one can make the second quantization to the Hamiltonian ${H}_{ep}={E}_{a}\nabla \cdot {\bf{u}}$ which yields^29^

7 $${H}_{ep}={E}_{a}\sum _{n}\,\int d{q}_{z}{A}_{n}({q}_{z}^{2}+{q}_{l,n}^{2}){J}_{0}({q}_{l,n}r)({\hat{a}}_{n,{q}_{z}}{e}^{i{q}_{z}z}+{\hat{a}}_{n,{q}_{z}}^{\dagger }{e}^{-i{q}_{z}z}),$$

where ${\hat{a}}_{n,{q}_{z}}^{\dagger }$ and ${\hat{a}}_{n,{q}_{z}}$ are the creation and annihilation operators of the phonon of the *n*-th transverse mode and with longitudinal wave vector *q*_*z*_. The normalization factor reads ${A}_{n}=\tfrac{R}{2}\sqrt{\tfrac{\hslash }{\rho V{\omega }_{n}({q}_{z}){S}_{n}}}$. *ω*_*n*_(*q*_*z*_) is the phonon’s frequency. *ρ* and *V* are, respectively, the mass density and volume of the nanowire. *S*_*n*_ is given by

8 $$\begin{array}{rcl}{S}_{n} & = & \frac{{q}_{z}^{2}{R}^{2}}{2}[{J}_{1}{({q}_{l,n}R)}^{2}+{J}_{0}{({q}_{l,n}R)}^{2}]+\frac{{\beta }_{n}^{2}{q}_{t,n}^{2}{R}^{2}}{2}[{J}_{1}{({q}_{t,n}R)}^{2}+{J}_{0}{({q}_{t,n}R)}^{2}]\\  &  & +\frac{{q}_{l,n}^{2}{R}^{2}}{2}[{J}_{1}{({q}_{l,n}R)}^{2}-{J}_{0}({q}_{l,n}R){J}_{2}({q}_{l,n}R)]\\  &  & +\frac{{\beta }_{n}^{2}{q}_{z}^{2}{R}^{2}}{2}[{J}_{1}{({q}_{t,n}R)}^{2}-{J}_{0}({q}_{t,n}R){J}_{2}({q}_{t,n}R)]\\  &  & +\frac{2{\beta }_{n}{q}_{z}{q}_{l,n}R}{{q}_{l,n}^{2}-{q}_{t,n}^{2}}[{q}_{l,n}{J}_{0}({q}_{l,n}R){J}_{1}({q}_{t,n}R)-{q}_{t,n}{J}_{0}({q}_{t,n}R){J}_{1}({q}_{l,n}R)]\\  &  & -\frac{2{\beta }_{n}{q}_{z}{q}_{t,n}R}{{q}_{l,n}^{2}-{q}_{t,n}^{2}}[{q}_{t,n}{J}_{0}({q}_{l,n}R){J}_{1}({q}_{t,n}R)-{q}_{l,n}{J}_{0}({q}_{t,n}R){J}_{1}({q}_{l,n}R)]\,.\end{array}$$

Under the free-standing boundary condition, the coefficient *β*_*n*_ in this result is given by

9 $${\beta }_{n}=-\frac{2{q}_{z}{q}_{l,n}}{{q}_{t,n}^{2}-{q}_{z}^{2}}\frac{{J}_{1}({q}_{l,n}R)}{{J}_{1}({q}_{t,n}R)}.$$

Applying the Fermi’s golden rule, the rate of electron scattered by acoustic phonons between subband states (*i*, *k*_*z*_) and $(f,{k}_{z}^{^{\prime} })$ reads

10 $${W}_{if}({k}_{z},{k}_{z}^{^{\prime} })=\frac{2\pi }{\hslash }{|\langle {\psi }_{f}({k}_{z}^{^{\prime} })|{H}_{ep}|{\psi }_{i}({k}_{z})\rangle |}^{2}\delta [{E}_{f}({k}_{z}^{^{\prime} })-{E}_{i}({k}_{z})+\epsilon \hslash {\omega }_{n}({q}_{z})],$$

where *k*_*z*_ and ${k}_{z}^{^{\prime} }$ are, respectively, the initial and finial momentum of the electron. $\epsilon =\pm 1$ correspond to emission and absorption of a phonon (with momentum *q*_*z*_), while the condition of energy conservation is implied by the *δ* function.

In Eq. (10), the scattering matrix element is detailed as

11 $$\begin{array}{rcl}\langle {\psi }_{f}({k}_{z}^{^{\prime} })|{H}_{ep}|{\psi }_{i}({k}_{z})\rangle  & = & {E}_{a}\sum _{n}\,\sum _{\epsilon =\pm 1}\,\int d{q}_{z}{A}_{n}\frac{{[{\omega }_{n}({q}_{z})]}^{2}}{{v}_{l}^{2}}\frac{2\pi }{l}\delta \\  &  & \times ({k}_{z}-{k}_{z}^{^{\prime} }-\epsilon {q}_{z})\,{F}_{if}({q}_{l,n}){\hat{a}}_{n,{q}_{z}}^{\epsilon }.\end{array}$$

Here we have introduced ${\hat{a}}_{n,{q}_{z}}^{\pm 1}$ to denote ${\hat{a}}^{\dagger }$ and $\hat{a}$, respectively. We also introduced the reduced overlap integral as

12 $${F}_{if}({q}_{l,n})=\frac{2}{{J}_{L+1}({\alpha }_{m}^{L}){J}_{L^{\prime} +1}({\alpha }_{m^{\prime} }^{L^{\prime} })}{\int }_{0}^{1}\,d(\frac{r}{R})(\frac{r}{R}){J}_{L}(\frac{{\alpha }_{m}^{L}r}{R}){J}_{L^{\prime} }(\frac{{\alpha }_{m^{\prime} }^{L^{\prime} }r}{R}){J}_{0}({q}_{l,n}r).$$

The *δ*-function in Eq. (11) reflects the momentum conservation in the axial direction of the nanowire, while in deriving this result we used the method of *box normalization*, i.e., $\delta (k^{\prime} -k)=\delta \mathrm{(0)}=l/\mathrm{(2}\pi )$ when *k*′ = *k*.

Now, applying the formula $\delta [\varphi (x)]={\sum }_{k}\,\frac{\delta (x-{x}_{k})}{\varphi ^{\prime} ({x}_{k})}$, we transform the *δ*-function of energy conservation in Eq. (10) as follows:

13 $$\begin{array}{l}\delta [{E}_{f}({k}_{z}^{^{\prime} })-{E}_{i}({k}_{z})+\epsilon \hslash {\omega }_{n}({q}_{z})]\\ \begin{array}{rcl} & = & \sum _{p}\,\delta ({q}_{z}-{\tilde{q}}_{z,p})/|\partial [{E}_{f}({k}_{z}+\epsilon {\tilde{q}}_{z,p})+\epsilon \hslash {\omega }_{n}({\tilde{q}}_{z,p})]/\partial {q}_{z}|.\\  & = & \sum _{p}\,\delta ({q}_{z}-{\tilde{q}}_{z,p})JDOS({k}_{z},{\tilde{q}}_{z,p}).\end{array}\end{array}$$

Here we have introduced the *joint density of states* of electron and phonon as

14 $$JDOS({k}_{z},{\tilde{q}}_{z,p})={|\partial [{E}_{f}({k}_{z}+\epsilon {\tilde{q}}_{z,p})+\epsilon \hslash {\omega }_{n}({\tilde{q}}_{z,p})]/\partial {q}_{z}|}^{-1}.$$

In the above two equations, ${\tilde{q}}_{z,p}$ is the root of the algebraic equation ${E}_{f}({k}_{z^{\prime} })-{E}_{i}({k}_{z})\pm \hslash {\omega }_{n}({q}_{z})=0$.

After these preparations, we obtain the final expression we used in this work for the momentum relaxation rate as

15 $$\begin{array}{rcl}{W}_{if}({k}_{z}) & = & \sum _{n,p,\epsilon =\pm 1}\,\int d{k}_{z}^{^{\prime} }{W}_{if}({k}_{z},{k}_{z}^{^{\prime} },{q}_{z},n)(1-\frac{{k}_{z}^{^{\prime} }}{{k}_{z}})\\  & = & \frac{{E}_{a}^{2}}{4\pi \rho {v}_{l}^{4}}\sum _{n,p,\epsilon }\,{S}_{n}^{-1}\frac{\varepsilon {\tilde{q}}_{z,p}}{{k}_{z}}{|{F}_{if}({q}_{l,n})|}^{2}{[{\omega }_{n}({\tilde{q}}_{z,p})]}^{3}\,JDOS\\  &  & \times ({k}_{z},{\tilde{q}}_{z,p})\,[{N}_{{\rm{p}}h}({\omega }_{n}({\tilde{q}}_{z,p}))+\mathrm{(1}+\epsilon )/2],\end{array}$$

*N*_ph_ is the average number of the thermal phonons, with frequency ${\omega }_{n}({\tilde{q}}_{z,p})$. The weighting factor $(1-{k}_{z}^{^{\prime} }/{k}_{z})$ is from a consideration of angle dependence of the scattering. That is, in the case of ${k}_{z}^{^{\prime} } < {k}_{z}$, the electron either emits a phonon with *q*_*z*_ > 0 or absorbs a phonon with *q*_*z*_ < 0, resulting thus in an increase of the momentum relaxation rate and decrease of the electron mobility. On the contrary, if ${k}_{z}^{^{\prime} } > {k}_{z}$, the momentum relaxation rate is reduced. Finally, we mention that in present study we omitted the Umklapp process.
